# Development of Highly Efficient Estrogen Receptor β-Targeted Near-Infrared Fluorescence Probes Triggered by Endogenous Hydrogen Peroxide for Diagnostic Imaging of Prostate Cancer

**DOI:** 10.3390/molecules28052309

**Published:** 2023-03-02

**Authors:** Pei He, Xiaofei Deng, Bin Xu, Baohua Xie, Wenting Zou, Haibing Zhou, Chune Dong

**Affiliations:** 1 Department of Gynecological Oncology, Zhongnan Hospital of Wuhan University, School of Pharmaceutical Sciences, Wuhan University, Wuhan 430071, China; 2 State Key Laboratory of Virology, Hubei Province Engineering and Technology Research Center for Fluorinated Pharmaceuticals, Key Laboratory of Combinatorial Biosynthesis and Drug Discovery (Wuhan University), Ministry of Education, Frontier Science Center for Immunology and Metabolism, School of Pharmaceutical Sciences, Wuhan University, Wuhan 430071, China

**Keywords:** estrogen receptor β, H_2_O_2_-triggered, near-infrared, prostate cancer, in vivo imaging

## Abstract

Hydrogen peroxide is one of the most important reactive oxygen species, which plays a vital role in many physiological and pathological processes. A dramatic increase in H_2_O_2_ levels is a prominent feature of cancer. Therefore, rapid and sensitive detection of H_2_O_2_ in vivo is quite conducive to an early cancer diagnosis. On the other hand, the therapeutic potential of estrogen receptor beta (ERβ) has been implicated in many diseases including prostate cancer, and this target has attracted intensive attention recently. In this work, we report the development of the first H_2_O_2_-triggered ERβ-targeted near-infrared fluorescence (NIR) probe and its application in imaging of prostate cancer both in vitro and in vivo. The probe showed good ERβ selective binding affinity, excellent H_2_O_2_ responsiveness and near infrared imaging potential. Moreover, in vivo and ex vivo imaging studies indicated that the probe could selectively bind to DU-145 prostate cancer cells and rapidly visualizes H_2_O_2_ in DU-145 xenograft tumors. Mechanistic studies such as high-resolution mass spectrometry (HRMS) and density functional theory (DFT) calculations indicated that the borate ester group is vital for the H_2_O_2_ response turn-on fluorescence of the probe. Therefore, this probe might be a promising imaging tool for monitoring the H_2_O_2_ levels and early diagnosis studies in prostate cancer research.

## 1. Introduction

In recent years, the therapeutic potential of estrogen receptor beta (ERβ) in breast cancer, prostate cancer, lung cancer, the nervous system, and bone tissue has been revealed, and this target has attracted more and more attention [1,2,3,4]. However, the fundamental research of ERβ is insufficient. The sub cellular distribution, subtype expression and the role of ERβ in different diseases need to be further confirmed [5,6]. Moreover, prostate cancer (PCa) is the second most common cancer and the fifth most common cause of cancer-related deaths in men worldwide [7,8]. Prostate cancer is closely related to age, and some signaling pathways involving reactive oxygen species (ROS) play an important role in the occurrence and progression of cancer with age [9]. At the same time, ERβ is an important target for the treatment of prostate cancer, and its expression level is different in normal prostate tissue, prostatic hyperplasia tissue, benign prostate cancer tissue, and high-grade prostate cancer tissue [10,11]. Therefore, the ERβ-targeted probe can be exploited to monitor the lesions and carcinogenesis of the prostate, so as to promote the development of the early diagnosis of prostate cancer. In order to enhance the targeting of probes to prostate cancer, environment-responsive ERβ-targeted probes can also be designed in virtue of tumor-specific micro environmental information.

Reactive oxygen species (ROS) are a group of reactive short-lived oxygen-containing species, including superoxide, monomorphic oxygen, hydrogen peroxide, hydroxyl radicals, and peroxyl radicals [12]. As one of the most important reactive oxygen species, H_2_O_2_ plays a vital role in maintaining protein folding, cell signaling, body defense, and cell respiration [13,14,15]. However, an increasing number of studies have suggested that the overproduction of H_2_O_2_ in vivo causes oxidative damage to cellular structures or biomolecules; abnormal levels of H_2_O_2_ in mitochondria have also been linked to serious diseases such as cardiovascular disease, neurodegenerative diseases, Alzheimer’s disease, and cancer [16]. ROS increase in the tumor microenvironment can either directly promote tumor growth through cell damage or indirectly promote tumor growth through inhibition of cytotoxic lymphocytes. A prominent feature is a dramatic increase in H_2_O_2_ content [17,18,19]. In general, endogenous H_2_O_2_ have always maintained a low level of about 20 nM, whereas in pathological areas, such as tumor cells and activated immune cells, the H_2_O_2_ level can reach 100 μM and rise two to three orders of magnitude compared with normal cells [20]. In addition, the production rate of H_2_O_2_ was 0.5 nM/10^4^ cell/h, which was much higher than that of normal cells [21]. With a high production of H_2_O_2_ in diseases such as cancer and inflammation, H_2_O_2_ has become a significant target. Many diagnostic reagents, prodrugs, drug delivery systems and fluorescent probes targeting H_2_O_2_ have been developed [22,23,24]. In past decades, a variety of fluorescent probes for the detection of H_2_O_2_ have been synthesized, most of which are designed based on the combination of H_2_O_2_-responsive groups and organic fluorophores, including ethers (thioether, seleneether, tellurium ether), ferrocene, phenylborate ester/phenylboronic acid (PBE/PBA), aromatic oxalate, and proline [25]. Phenylborate ester is the most common group among them, because of its high selectivity and responsiveness of H_2_O_2_ [26,27,28].

A small molecule fluorescent probe is an important component of optical imaging. Because of the advantages of simple operation, low detection limit, good selectivity, high sensitivity, and high spatial and temporal resolution, it has become one of the most powerful tools for biological targets studies [29]. Compared with traditional fluorescent probes, near-infrared region (NIR, 650–900 nm) fluorescent probes have the features such as a deeper penetration in tissues and the elimination of background fluorescence interference, which greatly promote the imaging of molecular processes in vivo [30]. In addition, the large Stokes shift is conducive to biological imaging, avoiding the effects of the adverse effects of excitation sources and self-absorption. Dicyanomethylene-4*H*-pyran and its derivatives (DCM dyes) have unique spectral characteristics, such as an emission wavelength in the near-infrared region, a large Stokes shift, excellent photo stability, and fluorescence emission performance [31,32,33]. In recent years, it has been widely regarded as an excellent bioimaging fluorophore [34,35]. Previously, our group firstly reported the ERβ-targeting NIR fluorescent probes and hypoxic response fluorescent probes that were based on the DCM-OH structure, which themselves are both ERβ ligands and small molecule probes of ERβ fluorophores, showing the good ERβ imaging ability in vitro and in vivo [36,37]. As mentioned above, an abnormal level of H_2_O_2_ caused by a ROS increase in the tumor microenvironment has become a significant target for the diagnosis and treatment of cancers. However, there is no report on the development of H_2_O_2_-responsive ERβ probe for prostate cancer imaging.

**Figure 1:**
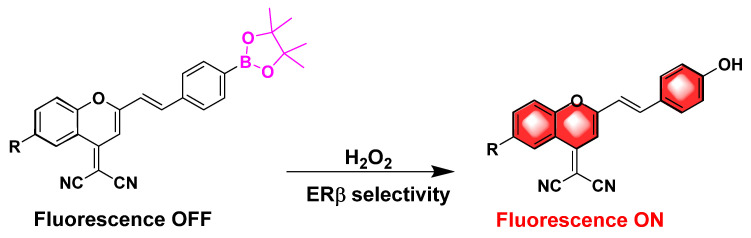
Design of ERβ-targeted, H_2_O_2_-triggered NIR probes for prostate cancer study.

As of now, H_2_O_2_ has been identified as a vital mediator of biological processes, in particular in cancer cell proliferation, differentiation, and migration; therefore, the development of a new H_2_O_2_-responsive probe could enable selective tracking of biological H_2_O_2_ fluxes and decipher new biological principles as well. As such, we designed and synthesized herein the first H_2_O_2_-responsive ERβ-targeted probes, which with DCM-OH form the skeleton of both the fluorophores and ERβ ligand, with borate esters as the H_2_O_2_-responsive group, thus trying to develop H_2_O_2_-triggered ERβ probes for prostate cancer imaging. We believe that the introduction of the borate ester group destroys the push–pull effect of the DCM-OH fluorophore and that the probe does not emit fluorescence. However, in the environment of a high concentration of H_2_O_2_, the borate ester group of the probe is specifically fractured by H_2_O_2_ and leaves, and is subsequently triggered and shows strong priming fluorescence and achieves tumor imaging in vitro and in vivo. Our study may bring new opportunities for prostate cancer diagnosis and research, which will certainly have greater practical application value (Figure 1).

## 2. Results and Discussion

### 2.1. Design and Synthesis of Probes

**Scheme 1:**
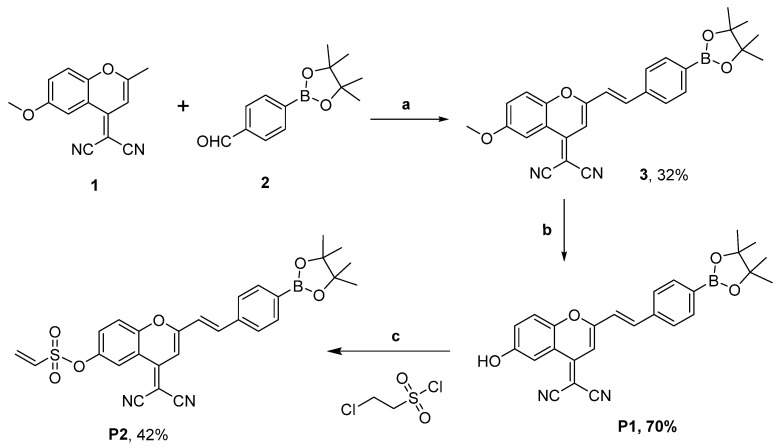
Synthetic route for probes **P1** and **P2**. Reagents and conditions: (a) Piperidine, CH_3_COOH, CH_3_CN, 85 °C, 12 h; (b) BBr_3_, DCM, 0 °C, overnight; (c) Et_3_N, DCM, rt.

The design and synthesis of probes was shown in Scheme 1; compound one was condensed with two in the presence of piperidine and acetic acid to obtain three. Next, the intermediate three was demethylated using boron tribromide to obtain probe **P1**. The probe **P2**, which contains an unsaturated alkene bond, was formed by reaction of **P1** with 2-chloroethane sulfonyl chloride. The intermediates and target compounds were confirmed with ^1^H NMR, ^13^C NMR, and high-resolution mass spectrometry. These data and detailed synthesis procedures for intermediates one and two (Scheme S1) are outlined in Supplementary Materials.

### 2.2. Optical Properties of Probes

In order to explore whether the target probe has animal imaging potential and verify the quenching effect of the borate ester group on fluorescence, we used PBS as the solvent to obtain the optical information of the probe (Table 1). The experimental results showed an obvious absorption peak of **P1** at 410 nm, and an NIR emission at 653 nm was observed. The emission wavelength has reached the near-infrared region, which has a good potential for animal imaging. The absorption peak of **P2** is also at 413 nm, and the NIR emission at 655 nm, which also meets the requirements of in vivo imaging. At the same time, it’s interesting that the Stokes shifts of **P1**–**P2** are more than 240 nm, and the anti-background interference ability is very strong. We tested the fluorescence quantum yield of the probe, and the results showed that the fluorescence quantum yield of **P1** and **P2** were low, because the introduction of the borate ester group effectively quenched the fluorescence of the DCM-OH parent nucleus. The possible reason may be that the DCM-OH fluorophore produces fluorescence mainly through the push and pull interaction between the cyano-group with strong electron absorption and the hydroxyl group with strong electron donation, while the introduction of the borate ester group destroys the push and pull interaction of the fluorescence parent nucleus, so the fluorescence quantum yield decreases.

**Figure 2:**
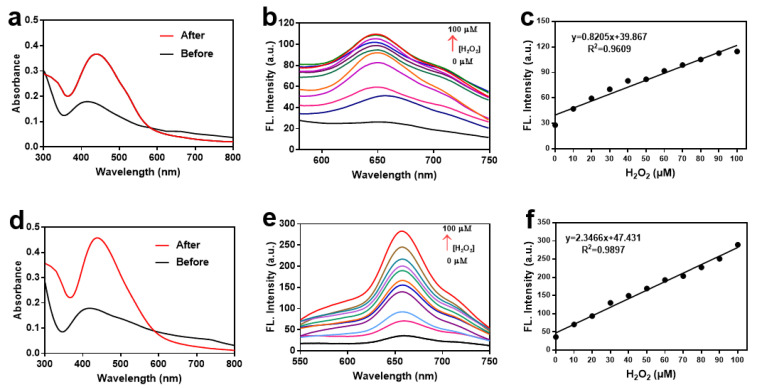
Absorption and fluorescence spectrum. (**a**) Absorption spectrum of **P1** (10 μM) before and after treatment with H_2_O_2_ in PBS. (**b**) Fluorescence spectrum of probe **P1** in concentration-dependent H_2_O_2_ (0–100 μM). (**c**) Linear relation spectrum of **P1** between fluorescence intensity and H_2_O_2_ concentration. (**d**) Absorption spectrum of **P2** (10 μM) before and after treatment with H_2_O_2_ in PBS. (**e**) Fluorescence spectrum of probe **P2** in concentration-dependent H_2_O_2_ (0–100 μM). (**f**) Linear relation spectrum of **P2** between fluorescence intensity and H_2_O_2_ concentration. Incubation time: 10 min.

To see whether the products of **P1** and **P2** after oxidation via H_2_O_2_ have better optical properties, we treated probes **P1** and **P2** with H_2_O_2_ (Figure 2). It showed that the absorption spectrum and fluorescence emission spectrum of the probe solution were greatly different before and after the addition of H_2_O_2_. After treatment with H_2_O_2_, the absorption spectra of **P1** and **P2** were redshifted, indicating that the properties of the probe changed before and after the H_2_O_2_ response (Figure 2a,d). From the fluorescence spectroscopy, the fluorescence intensity of **P1** and **P2** was relatively low and did not emit fluorescence in the 600–700 nm range before H_2_O_2_ was added. However, after treatment with H_2_O_2_, **P1** and **P2** produced fluorescence emission peaks in the 600–700 nm range significantly, indicating that the oxidation product has a good potential for in vivo imaging (Figure 2b,e). Moreover, in order to show the effect of H_2_O_2_ on **P1** and **P2** more intuitively, we also detected the relationship between the fluorescence emission spectrum of the probe and the different concentrations of H_2_O_2_ (Figure 2c,f). Interestingly, the results exhibited that after **P1** was oxidized by H_2_O_2_, the fluorescence intensity of the solution showed a linear relationship with the concentration in the range of 0–100 μM (R^2^ = 0.9609). Meanwhile, the fluorescence intensity of the **P2** solution also displayed a good linear relation with concentration in the range of 0–100 μM (R^2^ = 0.9897), which indicated that the H_2_O_2_ responsive probe had a high sensitivity to the concentration. In addition, we found that after the oxidation of probe **P1** with H_2_O_2_, the product was the same as probe P5 in our previous studies [36]. Comparing the fluorescence intensity of **P1** and P5 with the same concentration as shown in Figure S1, the fluorescence intensity of **P1** is very weak before adding H_2_O_2_, which is lower than that of probe P5. It is worth noting that after adding H_2_O_2_, the fluorescence intensity of **P1** is greatly enhanced, even higher than that of P5. This is consistent with the characteristics of the “turn-on” probes, which may have more imaging advantages than “inherent” probes.

### 2.3. Interference Resistance of Probe

**Figure 3:**
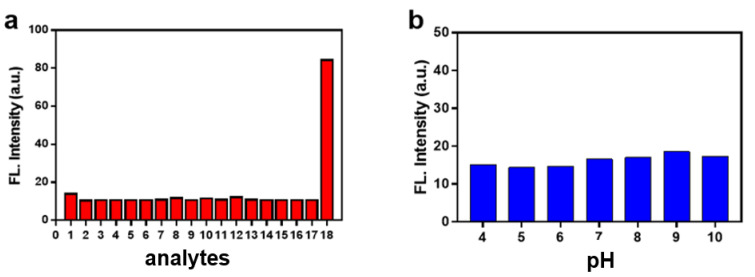
Fluorescence response of **P2** (10 μM) to various analytes and (**a**) different pH conditions (**b**). Groups were set as follows: 1, blank; 2, NaCl (100 mM); 3, MgCl_2_ (100 mM); 4, KCl (2.5 mM); 5, Tyr (1 mM); 6, GSH (1 mM); 7, His (1 mM); 8, Cys (1 mM); 9, Glu (1 mM); 10, Gly (1 mM); 11, Arg (1 mM); 12, NaOH (100 μM); 13, NaCO_3_ (100 μM); 14, Na_2_SO_4_ (100 μM); 15, NaNO_2_ (100 μM); 16, NaClO (100 μM); 17, NaCl (100 μM); 18, H_2_O_2_ (100 μM). Incubation time: 10 min.

There are various substances in the cell, such as anions, cations, amino acids, and reducing substances, which may all interfere with the experimental results. Therefore, **P2** was used to explore the anti-interference of the probe, and the H_2_O_2_-treated group was used as the positive control group (Figure 3a). After adding positive ions (Na^+^, Mg^+^, K^+^), common anions (OH^−^, CO_3_^2−^, SO_4_^2−^, NO_2_^−^, Cl^−^, ClO^−^), and amino acids (Tyr, GSH, His, Cys, Glu, Gly, Arg) to the probe for 60 min, no fluorescence increase was detected. Moreover, the fluorescence recovered after the addition of H_2_O_2_, indicating that common anions, cations, and amino acids had no significant interference on the detection of H_2_O_2_, so the probe would not be interfered by other substances when used for imaging in the physiological environment. We detected the effect of pH on probe fluorescence by adding **P2** separately to PBS solutions with a pH = 4–10 and then observing the fluorescence differences in the solutions (Figure 3b). The fluorescence intensity of **P2** changes little in different pH environments, which may be because the fluorescence of the probe is still in the quenched state, although the borate esters are sensitive to acid-base environments; the product of acid-base hydrolysis is boric acid. It can be seen that probes can be employed for imaging in physiological environments and are not affected under diverse pH conditions.

### 2.4. ERβ Selectivity of Probes

We first tested the *K*i values for ERα and ERβ of the probe (Table 2). The experimental results showed that the two probes displayed the same subtype selectivity. Due to the large borate ester group, they had a very weak affinity for ERα but had a certain affinity for ERβ. Compared with probe **P2**, probe **P1** has a lower binding affinity to ERβ. The ERβ selectivity of **P1** is three times higher than ERα. The addition of an unsaturated alkene double bond to the left side of probe **P1** increased the binding affinity of **P2** for ERβ with the corresponding *K*i value of 1.21 μM. Simultaneously, the ERβ selectivity of **P2** increased 7.75-fold more than ERα. This result is consistent with our expected assumption, indicating that probe **P2** is a potential ERβ-targeting fluorescent probe.

### 2.5. Imaging of Probe in Living Cells

To investigate the toxicity of **P1** and **P2** on normal cells and their inhibitory activity on cancer cells before performing cellular imaging, we tested the effects of **P1** and **P2** on MCF-10A normal breast cells, MCF-7 breast cancer cells with high ERα expression, and DU-145 prostate cancer cells with high ERβ expression by using CCK-8 assays (Table S1). The results showed that probe **P2** had no cytotoxicity to the MCF-10A normal cell line and DU-145 prostate cancer cell line, but it showed a certain inhibitory effect on the MCF-7 cancer cell line with an IC_50_ value of 16.34 ± 4.42 μM. However, **P1** was not toxic to any of these three cell lines. This indicated that our probe does not damage normal cells and tissues when used for imaging and can truly and accurately reflect the expression level of ERβ in the lesion area.

**Figure 4:**
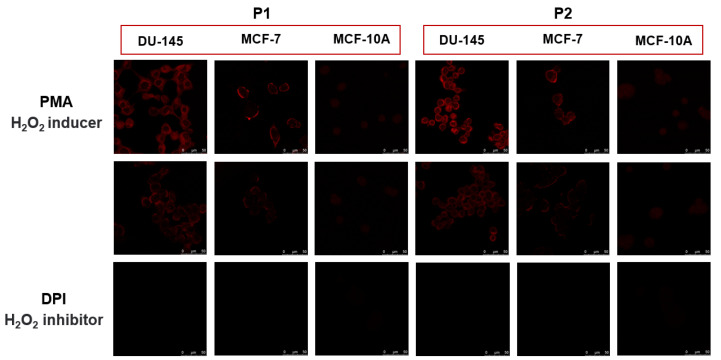
**P1**–**P2** (10 μM) showed responsiveness to H_2_O_2_ in MCF-10A, MCF-7, and DU-145 cells. PMA: H_2_O_2_ inducer; DPI: H_2_O_2_ inhibitor. Incubation time: 15 min. Excitation: 488 nm, emission: 600–700 nm.

In live cell imaging, we first explored their selectivity to ER isoforms and responsiveness to H_2_O_2_ with PMA as the H_2_O_2_ inducer and DPI as the H_2_O_2_ inhibitor (Figure 4). When the probe, probe + PMA, and probe + DPI were added to live cells 15 min later, weak fluorescence was observed in MCF-10A, MCF-7, and DU-145 cells without PMA, and more obvious fluorescence was observed in these two cells after added PMA, which indicated that **P1** and **P2** had a strong response to H_2_O_2_. In the H_2_O_2_ environment, the borate ester group was specifically removed, thus releasing fluorescence. At the same time, the inhibition of H_2_O_2_ production by DPI completely eliminated the fluorescence signal in MCF-10A, MCF-7, and DU-145 cells, and the cells shrunk and deformed, indicating that the consumption of H_2_O_2_ not only led to a decrease in the fluorescence intensity of the probe, but also had a great damage effect on cancer cells. This has reference significance for the development of bifunctional probes in the future. In addition, **P1** and **P2** are both targeted to ERβ; this targeting was more pronounced in cell imaging. The fluorescence signals of **P1** and **P2** were stronger in DU-145 cells with high ERβ expression, especially for probe **P2**. But they showed a weaker image in MCF-7 cells. In MCF-10A normal mammary epithelial cells, the fluorescence signal is particularly weak and almost invisible. Therefore, **P1** and **P2** have good selectivity towards ERβ and excellent H_2_O_2_ responsiveness, so it is expectable to obtain high ERβ subtype-targeted H_2_O_2_-responsive probes through structural modification.

**Figure 5:**
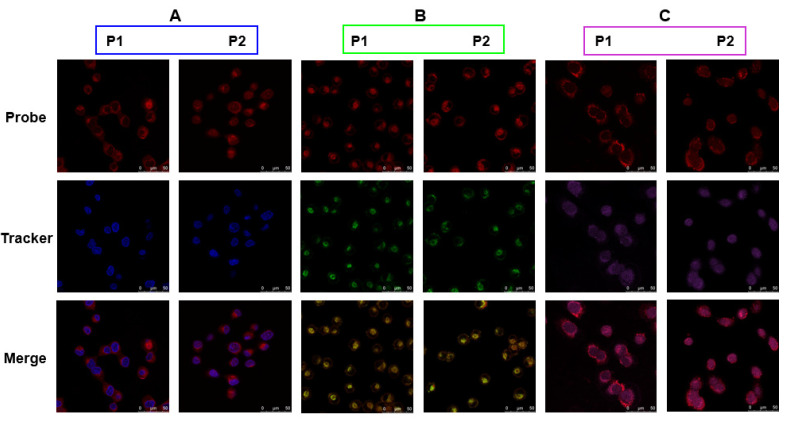
Probes colocalized imaging. **P1**–**P2** (10 μM) with nuclear dye DAPI (blue) (**A**), mitochondrial dye Mito-Tracker (green) (**B**), and ERβ secondary antibody dye (purple) (**C**), respectively, in DU-145 cells.

Subsequently, we investigated the colocalization of the probes and the nuclear dye DAPI in DU-145 cells (Figure 5A). The positions of **P1**, **P2**, and DAPI did not coincide in DU-145 cells which a high expression of ERβ; instead, they distributed in the extracellular region with a small overlap with the nuclear dye DAPI. We used Mito-Tracker Green, a mitochondrial dye, to study the distribution of probes in the cytoplasm (Figure 5B). Imaging results showed that the two probes were highly colocalized with mitochondria, and the co-location analysis diagrams were shown in Figure S2, which indicated that they were targeted to mitochondria. The ERβ labeling ability of the probes was further confirmed via immunofluorescence staining (Figure 5C). **P1** and **P2** were partially colocalized with ERβ, and the fluorescence signal mainly existed in the outer circle of the cell, which was similar to the colocalization result of mitochondria. Comparing the mitochondrial colocalization and immunofluorescence imaging results, it was proved that probes were targeting the mitochondrial ERβ (mtERβ), which has been identified by studies before [38,39]. Although it is reported that the mtERβ might be associated with apoptosis [40], the function of mtERβ in tumorigenesis remains unclear. At this juncture, probes **P1** and **P2** with a mtERβ targeting ability can be used as a new tool for the early diagnosis of prostate cancer.

### 2.6. In Vivo Imaging

**Figure 6:**
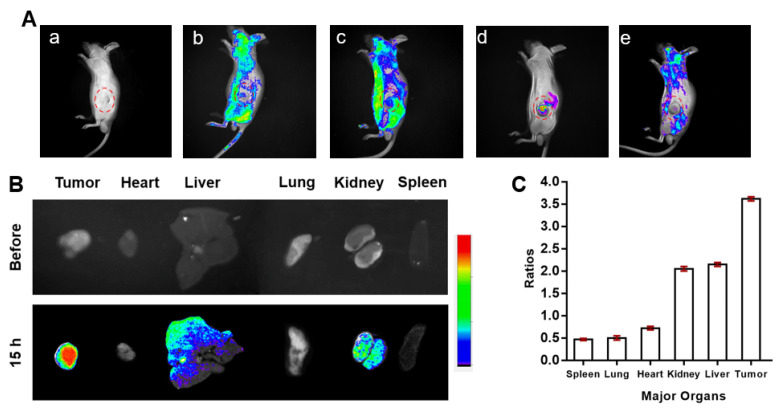
In vivo and ex vivo fluorescence imaging. (**A**) In vivo fluorescence imaging of **P2** (0.5 mg/kg) in DU-145 tumor-bearing mice at different time points (0 (**a**), 3 (**b**), 6 (**c**), 15 (**d**), and 36 h (**e**)). (**B**) Ex vivo fluorescence imaging of the tumor and major organs at 15 h after the injection of the probe. (**C**) Regions of interest (ROI) analysis of signal-to-background ratios.

As shown above, the good imaging ability of **P2**-targeting prostate cancer cells was more pronounced in vitro (Figure 4). Compared with **P1**, the fluorescence signal of **P2** was stronger in DU-145 prostate cancer cells with high ERβ expression; however, **P2** showed a weaker image in MCF-7 cells. Moreover, in normal MCF-10A mammary epithelial cells, the fluorescence signal of **P2** is particularly weak and almost invisible. In addition, according to colocalized imaging results in DU-145 prostate cancer cells, the ERβ labeling ability of the probe **P2** was further confirmed via immunofluorescence staining (Figure 5C). Considering the good imaging ability of probe **P2** to target prostate cancer cells in vitro, we further performed fluorescence imaging in vivo and then tracked the imaging process to determine whether it could be accurately visualized in a DU-145 xenograft mice model. After the injection of probe **P2** into the tail vein, representative fluorescence images and corresponding fluorescence intensity ratios of normal tissues to tumors at different time points (0, 3, 6, 15, and 36 h) are shown in Figure 6A. The probe did not show obvious fluorescence signal in nude mice at the beginning. Due to **P2** having the ability to target ERβ protein highly expressed in DU-145 xenograft tumors, the probe will gradually enrich in the tumor site. The fluorescence signal in the tumor area is stronger than the background in 15 h, showing good tumor targeting. The mild fluorescence signals were also observed in the liver region. It seems that there is a fluorescence signal in the mice brain. Chen’s group also found this signal in a related study, which might be because ERβ is also expressed in brain tissue [41]. Subsequently, we performed ex vivo animal imaging of mice to further determine the exact tissue distribution of probe **P2**. The fluorescence signal of tumor tissues was significantly stronger than that of the heart, liver, spleen, lung, kidney, and other tissues, but weaker fluorescence was also observed in the liver and kidney (Figure 6B,C), indicating that the probe may be metabolized by these two organs. The fluorescence signal of the kidney tissue could be observed to be significantly enhanced at 18 h, the fluorescence signal of tumor tissue and various organ tissues was basically weak at 24 h, and the probe was completely metabolized in mice at 36 h (Figure S3). **P2** exhibited excellent ERβ selectivity and good tumor targeting ability in vivo, which has important implication for imaging studies of ERβ prostate cancer.

### 2.7. The Mechanism of Response towards H_2_O_2_

**Scheme 2:**
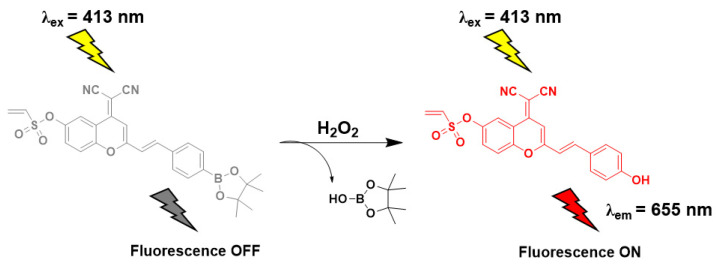
Proposal mechanism of the probe **P2** response to H_2_O_2_.

We hypothesized that since the introduction of borate ester groups destroys the push–pull effect of the DCM-OH fluorophore, the probe does not emit fluorescence, resulting in fluorescence quenching. However, when it entered DU-145 cells with a high concentration of reactive oxygen species, the borate ester group is specifically broken by H_2_O_2_ and leaves to become a phenolic hydroxyl group (electron-donating group), resulting in a strong fluorescence enhancement. Therefore, in order to reveal the mechanism of its fluorescence response, we analyzed the cell culture medium of **P2** using high-resolution mass spectrum (HRMS). The mass fragments observed at 529.1588 and 441.0522 belong to **P2** ([M + H]^+^, 529.1599) and **P2** + H_2_O_2_ ([M + Na]^+^, 441.0516), respectively (Figure S3), which well validated the proposed mechanism of action, the probe (Scheme 2).

**Figure 7:**
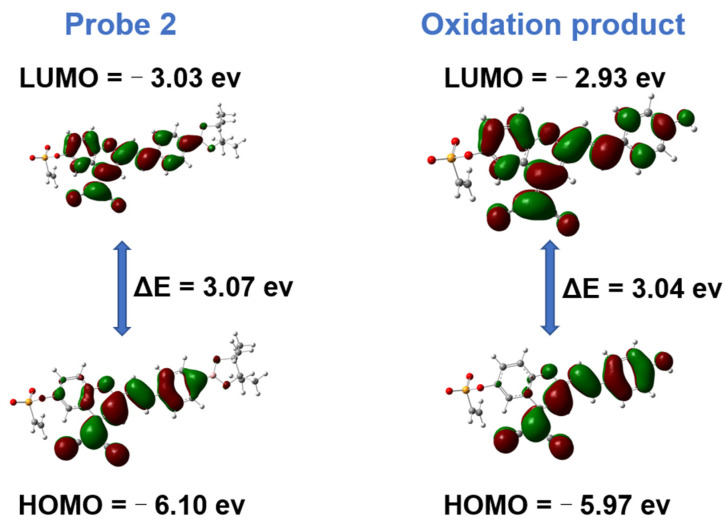
Density functional theory calculation for mechanistic study.

Finally, a DFT calculation was performed to analyze its turn-on mechanism from a theoretical perspective (Figure 7). According to the results of the lowest unoccupied molecular orbital (LUMO) energy and the highest occupied molecular orbital (HOMO) energy of probe **P2** and its H_2_O_2_ oxidating product, the electrons on the fluorophore are transferred to the borate ester group via a photoinduced electron transfer (PET) upon excitation, leading to fluorescence quenching. However, when the borate ester group is oxidized and broken into the hydroxyl group, the probe formed a D–π–A skeleton and released fluorescence, thus blocking the PET effect. In addition, the phenolic hydroxyl group can increase the intramolecular charge transfer (ICT) effect of the fluorophore and improve the fluorescence performance, which is consistent with the electron cloud change between the LUMO and HOMO of the oxidation product. In summary, the PET effect of borate ester group is the key to fluorescence quenching; the probe forms a D–π–A skeleton via oxidation to hydroxyl, realizing the fluorescence turn-on phenomenon, blocking the PET effect, and maintaining the ICT effect of fluorophore.

## 3. Materials and Methods

### 3.1. Materials and Measurements

The chemicals used in the experiments were purchased commercially. ^1^H NMR and ^13^C NMR spectra were obtained on a Bruker Biospin AV400 (400 MHz) (Billerica, MA, USA) instrument with TMS as the internal standard, and the compound charge/mass ratio was obtained using IonSpec 4.7 Tesla FTMS mass spectrometer (Irvine, CA, USA). The UV spectrum information of the probe was obtained via the SHIMADZU UV-2600 (Kyoto, Japan), and the fluorescence spectrum information of the probe was obtained via the HITACHI F-4600 (Hitachi, Japan). Cell imaging was performed using a LECA-LCS-SP8 laser confocal microscope (Wetzlar, Germany).

### 3.2. Chemical Synthesis

The synthetic route for the probes is based on the modification method described in references [42,43].

#### 3.2.1. (*E*)-2-(6-Methoxy-2-(4-(4,4,5,5-tetramethyl-1,3,2-dioxaborolan-2-yl)styryl)-4*H*-chromen-4-ylidene)malononitrile (**3**)

Under an argon atmosphere, compound 1 (500.0 mg, 2.1 mmol) and 2 (580 mg, 2.5 mmol) were added to the acetonitrile solution, then piperidine and acetic acid (piperidine: acetic acid = 1 mL: 0.5 mL) were added in turn and the reaction was heated to 85 °C and refluxed for 12 h. Then, it was extracted with dichloromethane (50 mL × 3), washed with saturated sodium chloride solution (20 mL × 1), and dried with anhydrous sodium sulfate; the organic phase was concentrated, and the crude product was purified using column chromatography (petroleum ether/ethyl acetate = 9:1) to obtain yellow solid **3** (300 mg). Yield: 32%. ^1^H NMR (400 MHz, Acetone-*d*_6_) δ 8.38 (d, J = 2.8 Hz, 1H), 7.76 (dd, J = 14.3, 12.7 Hz, 2H), 7.68 (d, J = 8.6 Hz, 2H), 7.52 (dd, J = 9.2, 2.9 Hz, 1H), 7.21 (d, J = 16.0 Hz, 1H), 6.95 (d, J = 8.6 Hz, 2H), 6.91 (s, 1H), 3.93 (s, 3H), 1.30 (s, 12H).

#### 3.2.2. (*E*)-2-(6-Hydroxy-2-(4-(4,4,5,5-tetramethyl-1,3,2-dioxaborolan-2-yl)styryl)-4*H*-chromen-4-ylidene)malononitrile (**P1**)

Three (300 mg, 0.66 mmol) was added to the anhydrous DCM solution under an ice bath filled with argon, dissolved and stirred, followed by the rapid addition of BBr_3_ (500.0 mg, 2.0 mmol), and stirred overnight under an ice bath. Then, it was quenched by slowly adding water, extracted with dichloromethane (30 mL × 3), washed with saturated sodium chloride solution (20 mL × 1), dried with anhydrous sodium sulfate; then, the organic phase was concentrated, and the crude product was purified using column chromatography (petroleum ether/ethyl acetate = 2:1) to afford orange-red solid **P1** (200 mg). (Yield: 70%, m. p. 225.5–225.8 °C). ^1^H NMR (400 MHz, DMSO-*d*_6_) δ 8.48 (s, 1H), 8.00 (d, J = 2.6 Hz, 1H), 7.68–7.60 (m, 2H), 7.58 (d, J = 8.7 Hz, 2H), 7.43 (d, J = 11.8 Hz, 1H), 7.15 (d, J = 15.9 Hz, 1H), 6.85 (s, 1H), 6.81 (d, J = 8.7 Hz, 2H), 1.70 (s, 12H). ^13^C NMR (125 MHz, DMSO-*d_6_*) δ 176.68 (s), 169.35 (s), 161.00 (s), 154.98 (s), 148.08 (s), 141.15 (s), 134.08 (s), 132.63 (s), 132.01 (s), 128.38 (s), 122.53 (s), 120.29 (s), 119.84 (s), 118.86 (s), 118.43 (s), 118.30 (s), 110.42 (s), 107.46 (s), 60.11 (s), 24.46 (s). HRMS (ESI) calcd. for C_26_H_23_BN_2_O_4_ [M + Na]^+^, 461.1643; found 461.1642.

#### 3.2.3. (*E*)-4-(Dicyanomethylene)-2-(4-(4,4,5,5-tetramethyl-1,3,2-dioxaborolan-2-yl)styryl)-4*H*-chromen-6-yl ethenesulfonate (**P2**)

Under an argon atmosphere, **P1** (200 mg, 0.46 mmol) was added to anhydrous DCM solution, dissolved and stirred, followed by 2-chloroethane sulfonyl chloride (90.0 mg, 0.55 mmol) and triethylamine (140.0 mg, 1.38 mmol), and stirred overnight at room temperature. Next, extracted with dichloromethane (30 mL × 3), washed with saturated sodium chloride solution (20 mL × 1), dried with anhydrous sodium sulfate; the organic phase was concentrated, and the crude product was purified via column chromatography (petroleum ether/ethyl acetate = 2:1) to obtain red solid **P2** (100 mg). (Yield: 42%, m. p. 200.8–201.5 °C). ^1^H NMR (400 MHz, Acetone-*d_6_*) δ 8.24 (d, J = 2.5 Hz, 1H), 7.76 (d, J = 16.0 Hz, 1H), 7.69–7.65 (m, 3H), 7.58 (d, J = 8.6 Hz, 1H), 7.48 (t, J = 2.1 Hz, 1H), 7.44 (dd, J = 8.8, 2.8 Hz, 1H), 7.28 (dd, J = 8.6, 2.5 Hz, 1H), 7.20 (d, J = 16.0 Hz, 1H), 6.95 (d, J = 8.5 Hz, 2H), 6.88 (s, 1H), 1.38 (s, 12H). ^13^C NMR (125 MHz, CD_3_OD) δ 177.97 (s), 158.99 (s), 153.22 (s), 147.73 (s), 146.30 (s), 139.14 (s), 129.84 (s), 129.46 (s), 126.49 (s), 124.52 (s), 123.82 (s), 119.64 (s), 118.22 (s), 117.08 (s), 116.10 (s), 115.74 (s), 115.25 (s), 113.32 (s), 105.72 (s), 104.68 (s), 61.62 (s), 25.53 (s). HRMS (ESI) calcd. for C_28_H_25_BN_2_O_6_S [M + H]^+^, 529.1599; found 529.1588.

### 3.3. Optical Properties

Fluorescence performance test preparation with 10 mM in PBS (pH = 7.4) probe solution, using an ultraviolet visible light spectrophotometer and HITACHI SHIMADZU UV-2600F-4600 fluorescence spectrophotometer instrument measuring the optical properties of the probe. Fluorescein (Φ_f1_ = 0.85) was used as a control, and the fluorescence quantum yields of **P1**–**P2** were calculated using the following formula:

Φ_(sample)_ = Φ_(standard)_ × (A_(standard)_/A_(sample)_) × (S_(sample)_/S_(standard)_) (1)

The meaning of the abbreviations: Φ: Fluorescence quantum yield; A (standard) and A (sample): absorption values of controls and probes at λ_em_; and S (standard) and S (sample): emission peak-to-peak areas of controls and probes. Slit width = 10/10 nm.

For the anti-interference experiment, analytes, including cations, anions, various amino acids, and reactive substances, were added to **P2** (10 μM) in PBS. The response was measured using a HITACHI F-4600 spectrophotometer (slit = 10/10 nm).

### 3.4. ER Binding Affinity Assay

The binding affinities of the probes to ERα and ERβ were determined via fluorescence polarimetry (FPA). In a 384-well plate, 20 μL of potassium phosphate buffer consisting of 0.8 μM ERα or ERβ protein, 150 nM fluorescent ligand, and 2.4 μg bovine immunoglobulin was added, followed by 20 μL of the target compound solution and incubated for 2 h at 25 °C in the dark. Fluorescence polarization values were obtained with a citation 3 microplate reader (Biotek, Winooski, VT, USA) and the experimental results were analyzed and the *K*_i_ value of each compound was calculated.

### 3.5. CCK-8 Assay

Normal breast cells MCF-10A and prostate cancer cells DU-145 (purchased from ATCC, Rockville, MD, USA), breast cancer cells MCF-7 (purchased from the Type Culture Collection of the Chinese Academy of Sciences, Shanghai, China) were cultured in a phenol red DMEM liquid medium supplemented with 10% fetal bovine serum. When the cell density reached 80% to 90%, the cells were digested, and the cell suspension was spread into 96-well cell culture plates with a phenol red-free DMEM medium containing 10% CS. After the cells were completely adherent, the original culture medium was discarded, and 100 μL of fresh compound solution prepared with the DMEM medium containing 10% CS were added to each well. The concentration gradient of the compound is: 1 × 10^−7.5^ M, 1 × 10^−7^ M, 1 × 10^−6.5^ M, 1 × 10^−6^ M, 1 × 10^−5.5^ M, 1 × 10^−5^ M, 1 × 10^−4.5^ M, 1 × 10^−4^ M. After 4 days of drug-treated culture, the culture plate was removed, the culture medium was aspirated, 100 μL CCK-8 working solution was added to each well and incubated for 1.5–2 h at 37 °C in a 5% CO_2_ incubator. The plate was read on the microplate reader, the wavelength at 450 nm was selected as the reference wavelength, the experimental results were analyzed, and the IC_50_ was calculated.

### 3.6. Cell Imaging

The DMEM medium containing the above cells was placed in a cell incubator at 37 °C, and after resuscitation, the cells were transferred to confocal small dishes and cultured for 24 h. The probes (10 μM), probe + PMA (10 μM), and probe + DPI (10 μM) were added to the small dishes to stain the viable cells for 15 min, and then the cells were carefully washed with PBS buffer 3 times. In the colocalization experiment, the cells in the confocal dishes were stained with probes for 15 min, then washed with PBS buffer 3 times, fixed with 4% paraformaldehyde, permeated with 0.2% Triton X-100, and washed with PBS after 10 min. DAPI was added to stain nuclei or the Mito-tracker Green was added to stain mitochondria. After 30 min, the free dye was washed off with PBS to observe the imaging results. In immunofluorescence staining, the DU-145 cells were stained with a probe (10 μM) for 30 min, and then fixed and permeated through the membrane. The cells were washed three times with PBS and incubated with a monoclonal anti-ERβ antibody (1:200) for 12 h at 37 °C. The cells were washed with PBS, and the secondary antibody dye DyLight 488 AffiniPure Goat Anti-Rabbit IgG (1:200) was added for ERβ staining for 1 h, after washing off the free dye to observe the image. Imaging results were obtained with a Leica-lcs-sp8 confocal laser scanning microscope.

### 3.7. Animal Imaging

In the animal imaging study, the mouse DU-145 tumor transplantation model was established. Firstly, the prostate cancer cell DU-145 was expanded and cultured, then injected subcutaneously into the waist of 6-week-old male Balb/c nude mice (purchased from Beijing Vital River Laboratory Animal Technology Co., Ltd., Beijing, China). When the tumor had grown to approximately 80–100 mm^3^ in size, the probe was dissolved in PBS and injected into the mice through the tail vein (*n* = 3). After 30 min, the mice were anesthetized with 2% sodium pentobarbital and imaged with a living animal imager (Bruker Xtreme BI, Karlsruhe, Germany), and the imaging results were observed at 3-h intervals. Excitation: 510 nm, emission 700 nm.

### 3.8. DFT Calculation

All DFT theoretical calculations have been carried out using the Gaussian 09 D0.1 program package. The B3LYP density functional method with the D3(BJ) dispersion correction was employed in this work to carry out all the computations [44]. The 6–31G (d,p) basis set was used for the atoms in geometry optimizations. Vibrational frequency analyses at the same level of theory were performed on all optimized structures to characterize stationary points as local minima [45].

## 4. Conclusions

In conclusion, the first ERβ-targeting and H_2_O_2_-triggered turn-on fluorescent probes were successfully developed in this study. As expected, probes **P1** and **P2** possessed favorable ERβ binding affinity and high ERβ selectivity. In addition, they exhibited good optical properties and excellent imaging ability, including a quick turn-on fluorescence response to H_2_O_2_ and mitochondrial ERβ imaging ability. Most importantly, **P2** was able to selectively bind to prostate cancer DU-145 cells and displayed no toxicity toward normal cells. In the in vivo imaging studies, probe **P2** could rapidly and precisely identify tumor tissues in DU-145 xenograft tumors. We believe that these novel ERβ-targeted H_2_O_2_-triggered fluorescent probes will be useful for early prostate cancer diagnosis and therapy. These results implied that our study might be useful for investigating the role of mitochondrial ERβ in cancer, cardiovascular diseases, and neurological diseases, etc.

## Figures and Tables

**Table 1:** The absorption wavelength, emission wavelength, and Stokes shift of probes.

Cmpd.	λ_ex_^a^ (nm)	λ_em_^b^ (nm)	Stokes Shift (nm)	ε	Φ_fl_^c^
**P1**	410	653	243	1.41 × 10^4^	0.33
**P2**	413	655	242	1.82 × 10^4^	0.37

^a^ λ_ex_ is the maximum absorption wavelength. ^b^ λ_em_ is the emission wavelength. ^c^ Φ_fl_ is the relative fluorescence quantum yield (Fluorescein, Φ_fl_ = 0.85, as the control).

**Table 2:** The *K*_i_ values of **P1**–**P2** for ERα and ERβ.

Cmpd.	Structure	*K*_i_ (μM) ^a^		
		ERα	ERβ	β/α Ratio ^b^
**P1**	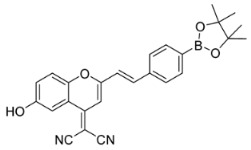	4.36 ± 0.01	1.42 ± 0.02	3
**P2**	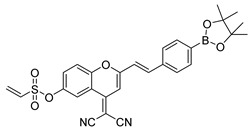	8.73 ± 0.01	1.21 ± 0.03	7.75

^a^*K*_i_ values are the mean ± SD of two or more experiments. The *K*_d_ value of estradiol is 3.49 nM for ERα and 4.12 nM for ERβ. ^b^ In each case, β/α ratios represent the affinity preference for ERβ vs. ERα.

## Data Availability

Data are contained within the article.

## Supplementary Materials

The following supporting information can be downloaded at: https://www.mdpi.com/article/10.3390/molecules28052309/s1, Scheme S1: Synthetic pathway of intermediates **1** and **2**. Figure S1: fluorescence intensity of **P1**, **P1** + H_2_O_2_, **P5**, **P5** + H_2_O_2_ (10 μM) in same concentration. Table S1: cell inhibitory activity of probes **P1** and **P2**. Figure S2: colocalization analysis of **P1** and **P2** in DU-145 cells. Figure S3: ex vivo fluorescence imaging for metabolism of probe **P2** in the tumor and major organs at 18 h, 24 h, 36 h. Figure S4: HMRS spectra of cell culture solution incubated with **P2**. Figure S5: HMRS spectra of **P1**. Figure S6: HMRS spectra of **P2**. Figures S7–S10: ^1^H NMR spectrum of **1b**, **1c**, **2a**, **1d**. Figures S11–S12: ^1^H NMR and ^13^C NMR spectrum of **P1**. Figures S13–S14: ^1^H NMR and ^13^C NMR spectrum of **P2**.

## Sample Availability

Samples of the compounds are available from the authors.

## References

[1] Kuiper G.G.J.M., Enmark E., PeltoHuikko M., Nilsson S., Gustafsson J.A. (1996). Cloning of a novel estrogen receptor expressed in rat prostate and ovary. Proc. Natl. Acad. Sci. USA.

[2] Katzenellenbogen B.S., Katzenellenbogen J.A. (2000). Estrogen receptor transcription and transactivation Estrogen receptor alpha and estrogen receptor beta: Regulation by selective estrogen receptor modulators and importance in breast cancer. Breast Cancer Res..

[3] Liu R., Xu X.L., Liang C.L., Chen X., Yu X.W., Zhong H.F., Xu W.X., Cheng Y., Wang W., Wu Y.D. (2019). ER beta modulates genistein’s cisplatin-enhancing activities in breast cancer MDA-MB-231 cells via P53-independent pathway. Mol. Cell. Biochem..

[4] Shen K., Yu H., Xie B., Meng Q., Dong C., Shen K., Zhou H.-B. (2023). Anticancer or carcinogenic? The role of estrogen receptor β in breast cancer progression. Pharmacol. Ther..

[5] Goswami I., Coutermarsh-Ott S., Morrison R.G., Allen I.C., Davalos R.V., Verbridge S.S., Bickford L.R. (2017). Irreversible electroporation inhibits pro-cancer inflammatory signaling in triple negative breast cancer cells. Bioelectrochemistry.

[6] Piperigkou Z., Bouris P., Onisto M., Franchi M., Kletsas D., Theocharis A.D., Karamanos N.K. (2016). Estrogen receptor beta modulates breast cancer cells functional properties, signaling and expression of matrix molecules. Matrix Biol..

[7] Wang G.C., Zhao D., Spring D.J., DePinho R.A. (2018). Genetics and biology of prostate cancer. Gene Dev..

[8] Cole A.P., Chen X., Langbein B.J., Giganti F., Kasivisvanathan V., Emberton M., Moore C.M., Lipsitz S.R., Keating N.L., Trinh Q.D. (2022). Geographic Variability, Time Trends and Association of Preoperative Magnetic Resonance Imaging with Surgical Outcomes for Elderly United States Men with Prostate Cancer: A Surveillance, Epidemiology, and End Results-Medicare Analysis. J. Urol..

[9] Khandrika L., Kumar B., Koul S., Maroni P., Koul H.K. (2009). Oxidative stress in prostate cancer. Cancer Lett..

[10] Christoforou P., Christopoulos P.F., Koutsilieris M. (2014). The Role of Estrogen Receptor beta in Prostate Cancer. Mol. Med..

[11] Tong D.L. (2022). Selective estrogen receptor modulators contribute to prostate cancer treatment by regulating the tumor immune microenvironment. J. Immunother Cancer.

[12] Sies H., Jones D.P. (2020). Reactive oxygen species (ROS) as pleiotropic physiological signalling agents. Nat. Rev. Mol. Cell Biol..

[13] Ray P.D., Huang B.W., Tsuji Y. (2012). Reactive oxygen species (ROS) homeostasis and redox regulation in cellular signaling. Cell Signal..

[14] Stone J.R., Yang S.P. (2006). Hydrogen peroxide: A signaling messenger. Antioxid. Redox Signal..

[15] Wenzel P., Kossmann S., Munzel T., Daiber A. (2017). Redox regulation of cardiovascular inflammation—Immunomodulatory function of mitochondrial and Nox-derived reactive oxygen and nitrogen species. Free Radic. Biol. Med..

[16] Giorgio M., Trinei M., Migliaccio E., Pelicci P.G. (2007). Hydrogen peroxide: A metabolic by-product or a common mediator of ageing signals?. Nat. Rev. Mol. Cell Biol..

[17] Lippert A.R., De Bittner G.C.V., Chang C.J. (2011). Boronate Oxidation as a Bioorthogonal Reaction Approach for Studying the Chemistry of Hydrogen Peroxide in Living Systems. Acc. Chem. Res..

[18] Sacks D., Baxter B., Campbell B.C.V., Carpenter J.S., Cognard C., Dippel D., Eesa M., Fischer U., Hausegger K., Hirsch J.A. (2018). Multisociety Consensus Quality Improvement Revised Consensus Statement for Endovascular Therapy of Acute Ischemic Stroke. Int. J. Stroke.

[19] Galadari S., Rahman A., Pallichankandy S., Thayyullathil F. (2017). Reactive oxygen species and cancer paradox: To promote or to suppress?. Free Radic. Biol. Med..

[20] Fang H.P., Chen J., Lin L., Liu F., Tian H.Y., Chen X.S. (2019). A Strategy of Killing Three Birds with One Stone for Cancer Therapy through Regulating the Tumor Microenvironment by H_2_O_2_-Responsive Gene Delivery System. ACS Appl. Mater. Inter..

[21] Yang N., Xiao W.Y., Song X.J., Wang W.J., Dong X.C. (2020). Recent Advances in Tumor Microenvironment Hydrogen Peroxide-Responsive Materials for Cancer Photodynamic Therapy. Nano-Micro Lett..

[22] Wang C.C., Wang Y., Wang G.Y., Huang C.S., Jia N.Q. (2020). A new mitochondria-targeting fluorescent probe for ratiometric detection of H_2_O_2_ in live cells. Anal. Chim. Acta.

[23] Wu Y., Li Z.Y., Shen Y.M. (2019). A Novel ESIPT Phthalimide-Based Fluorescent Probe for Quantitative Detection of H_2_O_2_. ACS Omega.

[24] Morgan B., Van Laer K., Owusu T.N.E., Ezerina D., Pastor-Flores D., Amponsah P.S., Tursch A., Dick T.P. (2016). Real-time monitoring of basal H_2_O_2_ levels with peroxiredoxin-based probes. Nat. Chem. Biol..

[25] Ye H., Zhou Y., Liu X., Chen Y.B., Duan S.Z., Zhu R.Y., Liu Y., Yin L.C. (2019). Recent Advances on Reactive Oxygen Species-Responsive Delivery and Diagnosis System. Biomacromolecules.

[26] Xu Y.H., Shi W., Li H.Y., Li X.H., Ma H.M. (2019). H_2_O_2_-Responsive Organosilica-Doxorubicin Nanoparticles for Targeted Imaging and Killing of Cancer Cells Based on a Synthesized Silane-Borate Precursor. ChemMedChem.

[27] Liu Y.Y., Jiao C.P., Lu W.J., Zhang P.P., Wang Y.F. (2019). Research progress in the development of organic small molecule fluorescent probes for detecting H_2_O_2_. RSC Adv..

[28] Wang Y.B., Fan H.L., Balakrishnan K., Lin Z.C., Cao S., Chen W.B., Fan Y.K., Guthrie Q.A., Sun H.B., Teske K.A. (2017). Hydrogen peroxide activated quinone methide precursors with enhanced DNA cross-linking capability and cytotoxicity towards cancer cells. Eur. J. Med. Chem..

[29] Pramanik S.K., Das A. (2021). Fluorescent probes for imaging bioactive species in subcellular organelles. Chem. Commun..

[30] Chu T.S., Lu R., Liu B.T. (2016). Reversibly monitoring oxidation and reduction events in living biological systems: Recent development of redox-responsive reversible NIR biosensors and their applications in in vitro/in vivo fluorescence imaging. Biosens. Bioelectron..

[31] Yuan L., Lin W.Y., Zheng K.B., He L.W., Huang W.M. (2013). Far-red to near infrared analyte-responsive fluorescent probes based on organic fluorophore platforms for fluorescence imaging. Chem. Soc. Rev..

[32] Wang J.F., Li B., Zhao W.Y., Zhang X.F., Luo X., Corkins M.E., Cole S.L., Wang C., Xiao Y., Bi X.M. (2016). Two-Photon Near Infrared Fluorescent Turn-On Probe Toward Cysteine and Its Imaging Applications. ACS Sens..

[33] Xiong J.C., Xia L.L., Li L.S., Cui M.Y., Gu Y.Q., Wang P. (2019). An acetate-based NIR fluorescent probe for selectively imaging of hydrogen peroxide in living cells and in vivo. Sens. Actuators B-Chem..

[34] Wang S., Ren W.X., Hou J.T., Won M., An J., Chen X.Y., Shu J., Kim J.S. (2021). Fluorescence imaging of pathophysiological microenvironments. Chem. Soc. Rev..

[35] Han H.H., Tian H., Zang Y., Sedgwick A.C., Li J., Sessler J.L., He X.P., James T.D. (2021). Small-molecule fluorescence-based probes for interrogating major organ diseases. Chem. Soc. Rev..

[36] Meng Q.Y., Xie B.H., Yu H.G., Shen K., Deng X.P., Zhou H.B., Dong C.N. (2022). Estrogen Receptor beta-Targeted Near-Infrared Inherently Fluorescent Probe: A Potent Tool for Estrogen Receptor beta Research. ACS Sens..

[37] Xie B.H., Meng Q.Y., Yu H.G., Shen K., Cheng Y., Dong C.E., Zhou H.B. (2022). Estrogen receptor beta-targeted hypoxia-responsive near-infrared fluorescence probes for prostate cancer study. Eur. J. Med. Chem..

[38] Liao T.-L., Tzeng C.-R., Yu C.-L., Wang Y.-P., Kao S.-H. (2015). Estrogen receptor-β in mitochondria: Implications for mitochondrial bioenergetics and tumorigenesis. Ann. N. Y. Acad. Sci..

[39] Klinge C.M. (2020). Estrogenic control of mitochondrial function. Redox Biol..

[40] Yang S.-H., Sarkar S.N., Liu R., Perez E.J., Wang X., Wen Y., Yan L.-J., Simpkins J.W. (2009). Estrogen receptor β as a mitochondrial vulnerability factor. J. Biol. Chem..

[41] Zhou Y., Lei P., Han J., Wang Z., Ji A., Wu Y., Zheng L., Zhang X., Qu C., Min J. (2023). Development of a Novel 18F-Labeled Probe for PET Imaging of Estrogen Receptor β. J. Med. Chem..

[42] Wang W.X., Jiang W.L., Liu Y., Li Y.F., Zhang J., Li C.Y. (2020). Near-infrared fluorescence probe with a large stokes shift for visualizing hydrogen peroxide in ulcerative colitis mice. Sens. Actuators B-Chem..

[43] Men J.X., Yang X.J., Zhang H.B., Zhou J.P. (2018). A near-infrared fluorescent probe based on nucleophilic substitution-cyclization for selective detection of hydrogen sulfide and bioimaging. Dyes Pigment.

[44] Grimme S., Ehrlich S., Goerigk L. (2011). Effect of the Damping Function in Dispersion Corrected Density Functional Theory. J. Comput. Chem..

[45] Rassolov V.A., Ratner M.A., Pople J.A., Redfern P.C., Curtiss L.A. (2001). 6-31G*basis set for third-row atoms. J. Comput. Chem..

