# Supplementary material for: Development of Highly Efficient Estrogen Receptor β-Targeted Near-Infrared Fluorescence Probes Triggered by Endogenous Hydrogen Peroxide for Diagnostic Imaging of Prostate Cancer
Source: Molecules. 2023 Mar 2;28(5):2309. doi: 10.3390/molecules28052309 (PMC10005547; doi:10.3390/molecules28052309)
Supplement: Supplementary file 1 [file molecules-28-02309-s001.zip › molecules-2230785-supplementary.pdf]

Supplementary Materials

Development of Highly Efficient Estrogen Receptor β-Targeted Near-Infrared Fluorescence Probes Triggered by Endogenous Hydrogen Peroxide for Diagnostic Imaging of Prostate Cancer

Pei He [1], Xiaofei Deng [1], Bin Xu [1], Baohua Xie [2], Wenting Zou [1], Haibing Zhou [1,2,*] and Chune Dong [1,2,*]

[1] Department of Gynecological Oncology, Zhongnan Hospital of Wuhan University, School of Pharmaceutical Sciences, Wuhan University, Wuhan 430071, China
[2] State Key Laboratory of Virology, Hubei Province Engineering and Technology Research Center for Fluorinated Pharmaceuticals, Key Laboratory of Combinatorial Biosynthesis and Drug Discovery (Wuhan University), Ministry of Education, Frontier Science Center for Immunology and Metabolism, School of Pharmaceutical Sciences, Wuhan University, Wuhan 430071, China
* Correspondence: zhouhb@whu.edu.cn (H.-B.Z.); cdong@whu.edu.cn (C.D.)

1. The synthesis of intermediates 1 and 2.

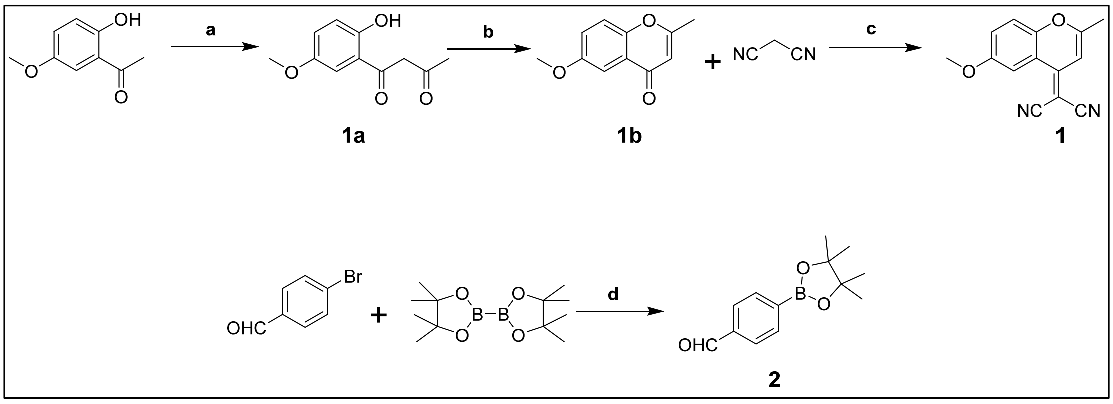

Scheme S1. Synthetic pathway of intermediates **1** and **2**. Reagents and conditions: (a) NaH, EtOAc, 0 °C, rt, 4 h; (b) H_2SO_4, CH_3COOH, 120 °C, 30 min; (c) Ac_2O, 140 °C, 14 h; (d) Pd(dppf)Cl_2, CH_3COOK, DMF, 80 °C, 3 h;

*1-(2-hydroxy-5-methoxyphenyl)butane-1,3-dione (**1a**)*

2'-Hydroxy-5-methoxy acetophenone (3.0 g, 18.0 mmol) was dissolved in 30 mL ethyl acetate, after dissolved and stirred, 60% NaH (3.0 g, 75.0 mmol) was slowly added in an ice bath, then removed the ice bath, and the mixture was stirred for 4 h at room temperature. Next, the NaH was quenched with ice water, and the pH was adjusted to neutral with 3 M HCl, then the solid was filtered and dried, gave a white crystalline solid **1a** (3.2 g, yield 85%).

*6-methoxy-2-methyl-4H-chromen-4-one (**1b**)*

1a (3.5g, 16.8mmol) was added to 40 mL CH_3COOH, and then 3.6 mL concentrated sulfuric acid was slowly added. After dissolved and stirred, the mixture was heated to 120 °C and reflux for 30 min. Then, slowly added the reaction solution to the ice water, add the saturated Na_2CO_3 solution and slowly adjust the pH to neutral. The mixture was extracted with dichloromethane (50 mL × 3), washed by saturated sodium chloride solution (20 mL × 1) and dried with anhydrous sodium sulfate. The organic layer was concentrated into a needle gray solid **1b** (2.4 g, 75% yield). ^{1}H NMR (400 MHz, $CDCl_3$) δ 7.49 (d, J = 3.1 Hz, 1H), 7.30 (d, J = 9.1 Hz, 1H), 7.18 (dd, J = 9.1, 3.1 Hz, 1H), 6.11 (s, 1H), 3.84 (s, 3H), 2.34 (s, 3H).

*2-(6-methoxy-2-methyl-4H-chromen-4-ylidene)malononitrile (**1**)*

1b (2.8 g, 14.7 mmol) and malononitrile (1.2 g, 18.2 mol) were added to 20 mL acetic anhydride, dissolved and stirred, and refluxed at 140 °C for 14 h. After cooling to room temperature, the reaction solution was concentrated in vacuum, then 30 mL deionized water was added, and the reaction was heated to reflux for 30 min. Next, it was cooled to room temperature, then extracted with dichloromethane (50 mL × 3), washed with saturated sodium chloride solution (20 mL × 1), and dried with anhydrous sodium sulfate, concentrated the organic phase, and purified by column chromatography (petroleum ether/ethyl acetate = 5:1) to obtain orange-red solid **1** (1.8g, yield 51%). ^{1}H NMR (400 MHz, $CDCl_3$) δ 8.35 (d, J = 2.8 Hz, 1H), 7.42 (d, J = 9.2 Hz, 1H), 7.33 (dd, J = 9.2, 2.8 Hz, 1H), 6.72 (s, 1H), 3.92 (s, 3H), 2.46 (s, 2H).

*4-(4,4,5,5-tetramethyl-1,3,2-dioxaborolan-2-yl)benzaldehyde (**2**)*

4-Bromobenzaldehyde (500.0 mg, 2.7 mmol), bis(pinacolato)diboron (1.4 g, 5.4 mmol), Pd(dppf)Cl_2 (200.0 mg, 0.27 mmol) and CH_3COOK (800.0 mg, 8.1mmol) were added to the reaction flask containing anhydrous DMF in sequence under an Ar atmosphere, dissolved and stirred, and then heated to 80 °C for 3 h. Next, extracted with dichloromethane (50 mL × 3), washed with saturated sodium chloride solution (20 mL × 1), and dried with anhydrous sodium sulfate, the organic phase was concentrated, and the crude product was purified by column chromatography (petroleum ether/ethyl acetate = 5:1) to afford white solid **2** (0.5g, 80%). ^{1}H NMR (400 MHz, $CDCl_3$) δ 10.03 (s, 1H), 7.95 (d, J = 6.8 Hz, 2H), 7.85 (d, J = 6.6 Hz, 2H), 1.35 (s, 12H).

2. Optical Properties of Probes.

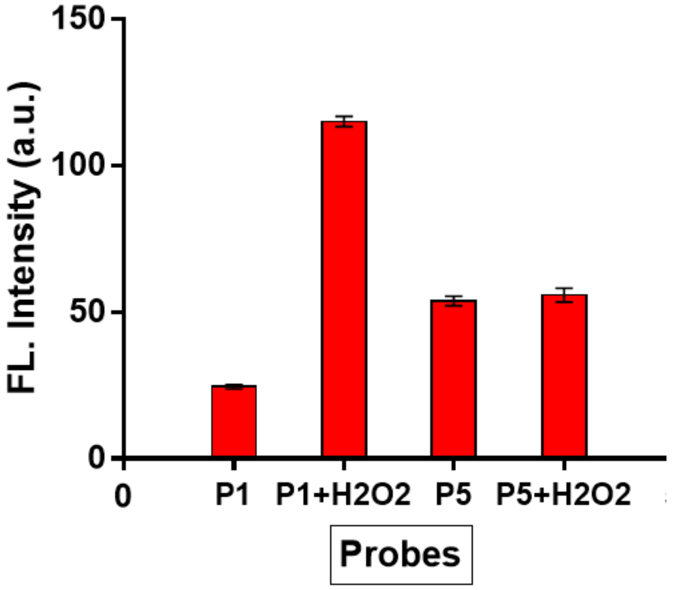

Figure S1. Fluorescence intensity of P1, P1+H_2O_2, P5, P5+H_2O_2 (10 μM) in same concentration.

3. Cytotoxicity.

Table S1. Cell inhibitory activity of probes **P1** and **P2**.

Cmpd.	MCF-10A (IC_{50}, μM)	MCF-7 (IC_{50}, μM)	DU-145 (IC_{50}, μM)
**P1**	> 100	> 100	> 100
**P2**	> 100	16.34 ± 4.42	> 100

4. Cell imaging.

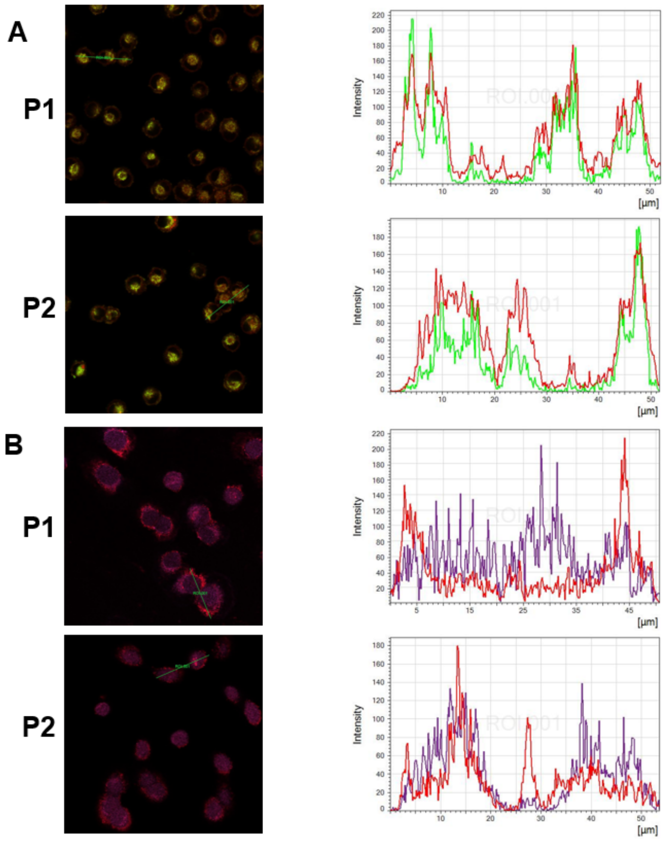

Figure S2. Colocalization analysis of **P1** and **P2** in DU-145 cells.

(A) Intensity profile of linear plot analysis of ROI (the brightest area) across DU-145 cells, which were co-stained by **P1-P2** and Mito-tracker Green.
(B) Intensity profile of linear plot analysis of regions of interest (ROI) (the brightest area) across DU-145 cells, which were co-stained by **P1-P2** and ERβ antibody dye.

5. *In vivo* imaging.

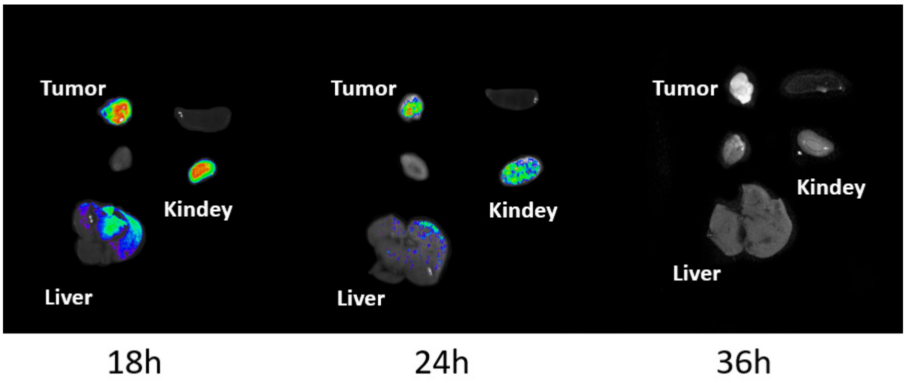

Figure S3. *Ex vivo* fluorescence imaging for metabolism of probe **P2** in the tumor and major organs at 18 h, 24 h, 36 h.

6. Mechanistic study.

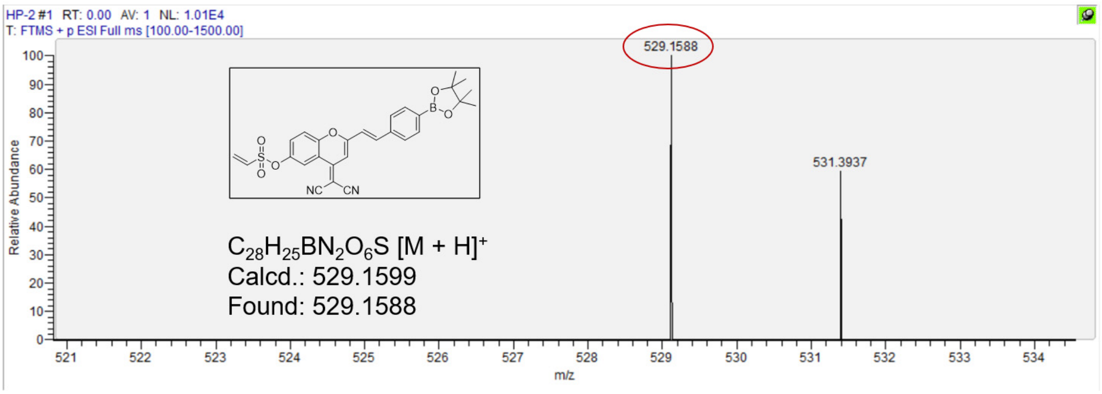

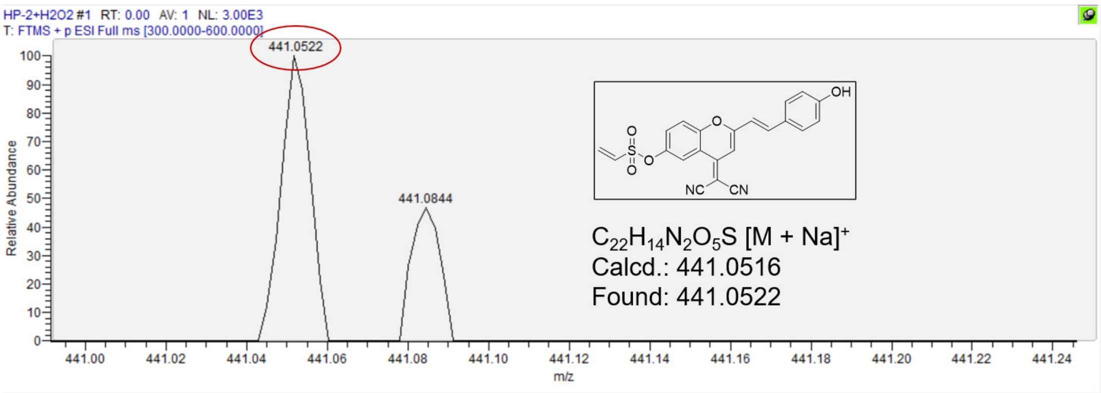

Figure S4. HMRS spectra of cell culture solution incubated with **P2**.

7. High-resolution mass spectra of P1 and P2.

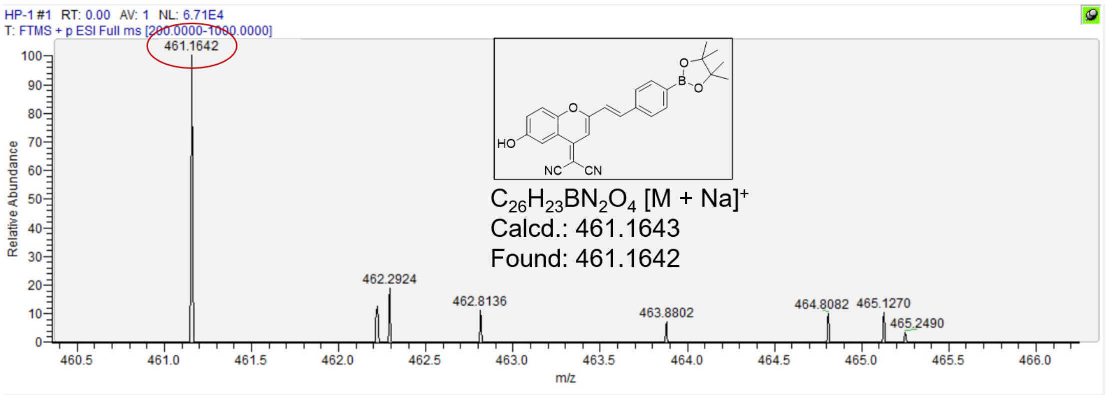

Figure S5. HMRS spectra of **P1**.

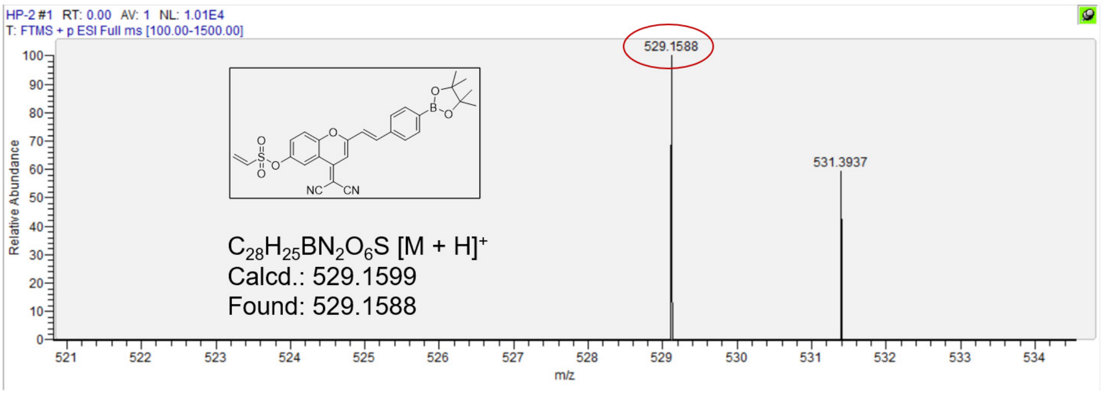

Figure S6. HMRS spectra of **P2**.

8. Spectra of ^{1}H and ^{13}C NMR.

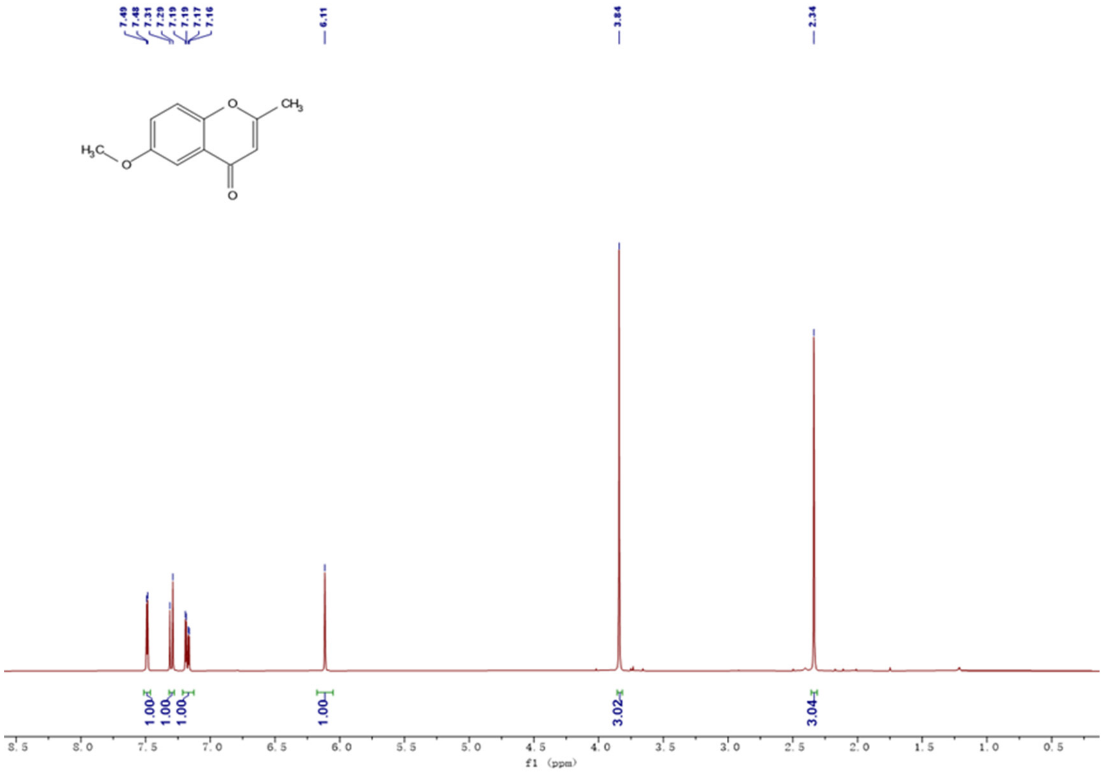

Figure S7. 1H NMR spectrum of **1b** in CDCl3.

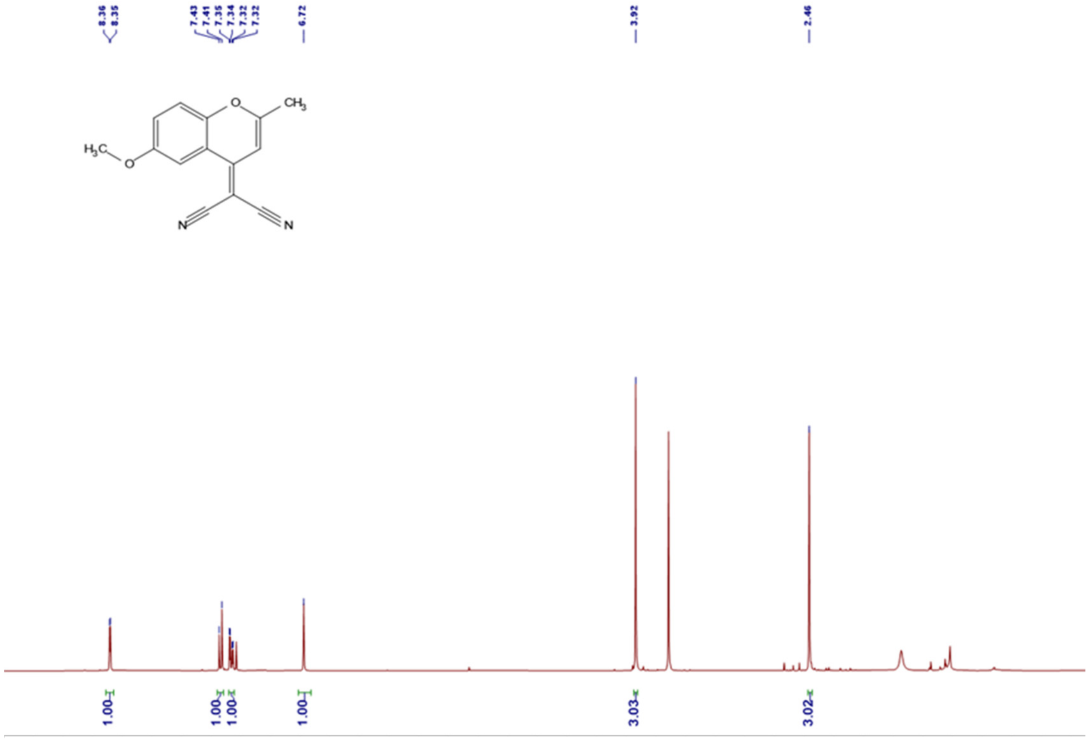

Figure S8. ^{1}H NMR spectrum of **1c** in CDCl3.

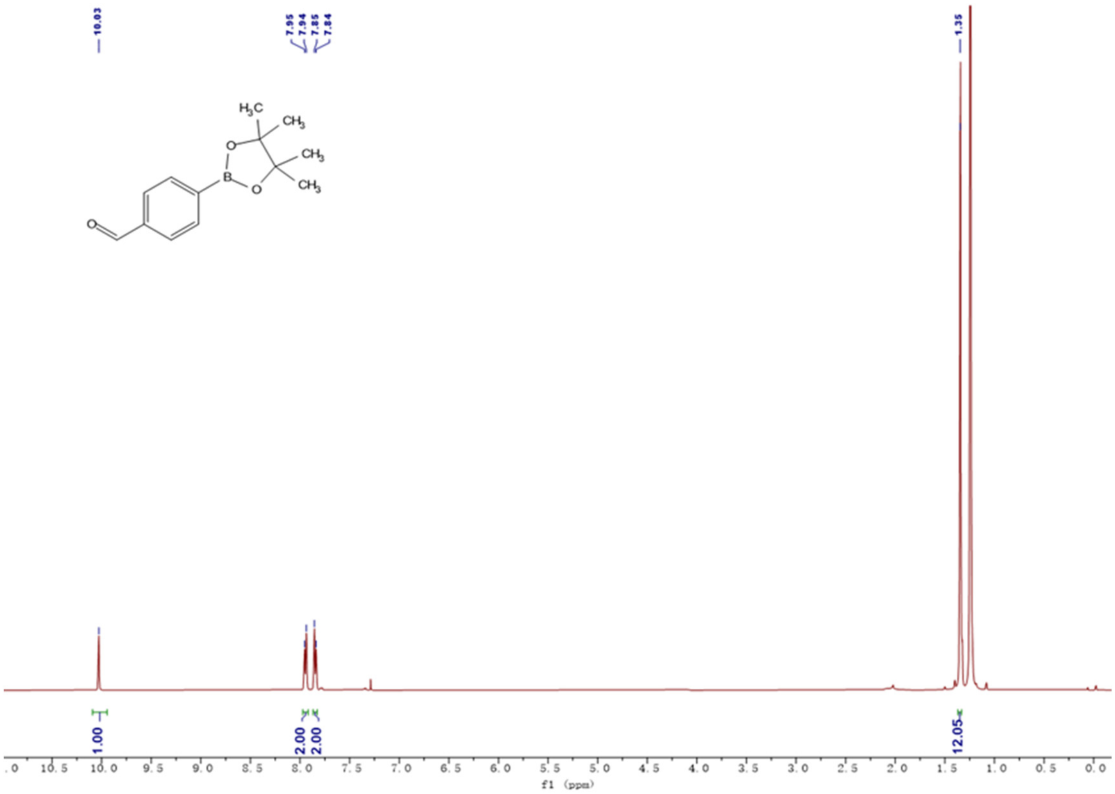

Figure S9. ^{1}H NMR spectrum of **2a** in $CDCl_3$.

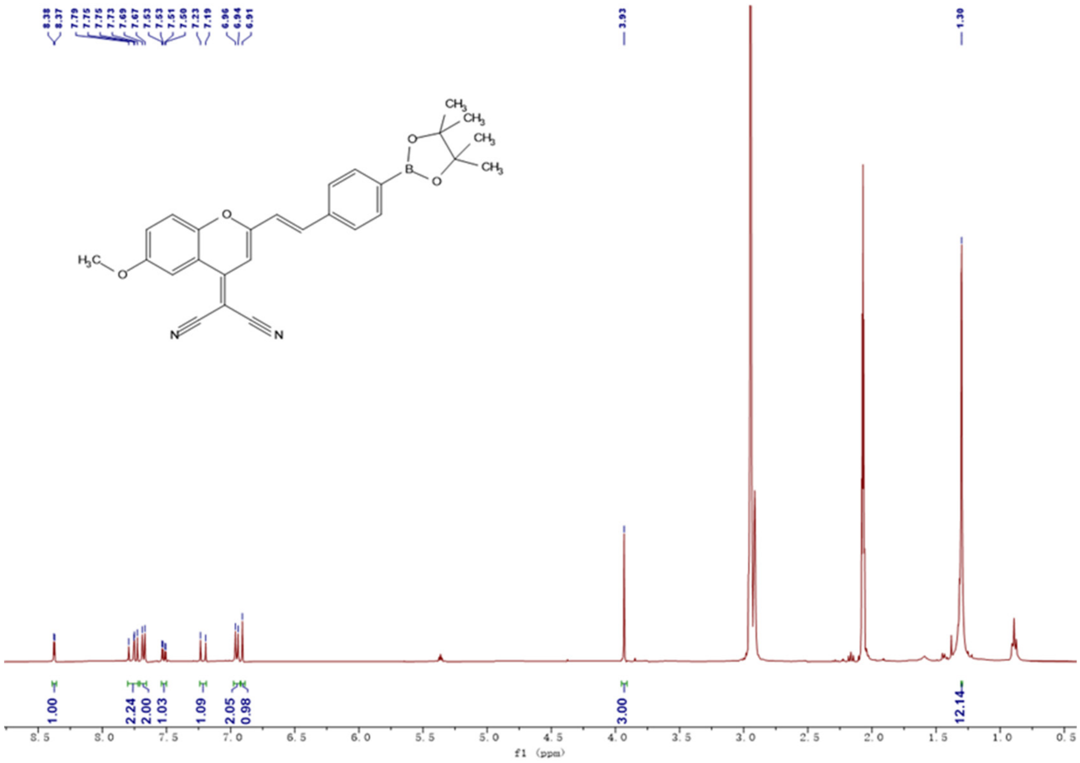

Figure S10. ^{1}H NMR spectrum of **1d** in Acetone-d_6.

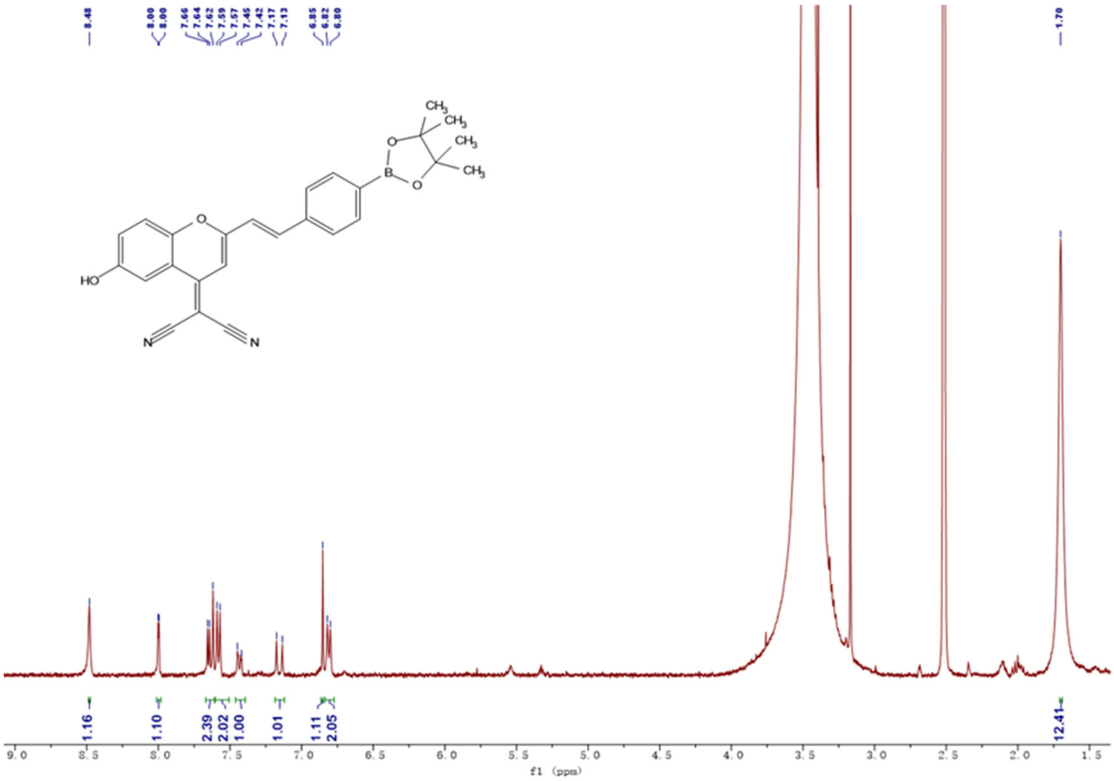

Figure S11. ^{1}H NMR spectrum of **P1** in DMSO-d_6.

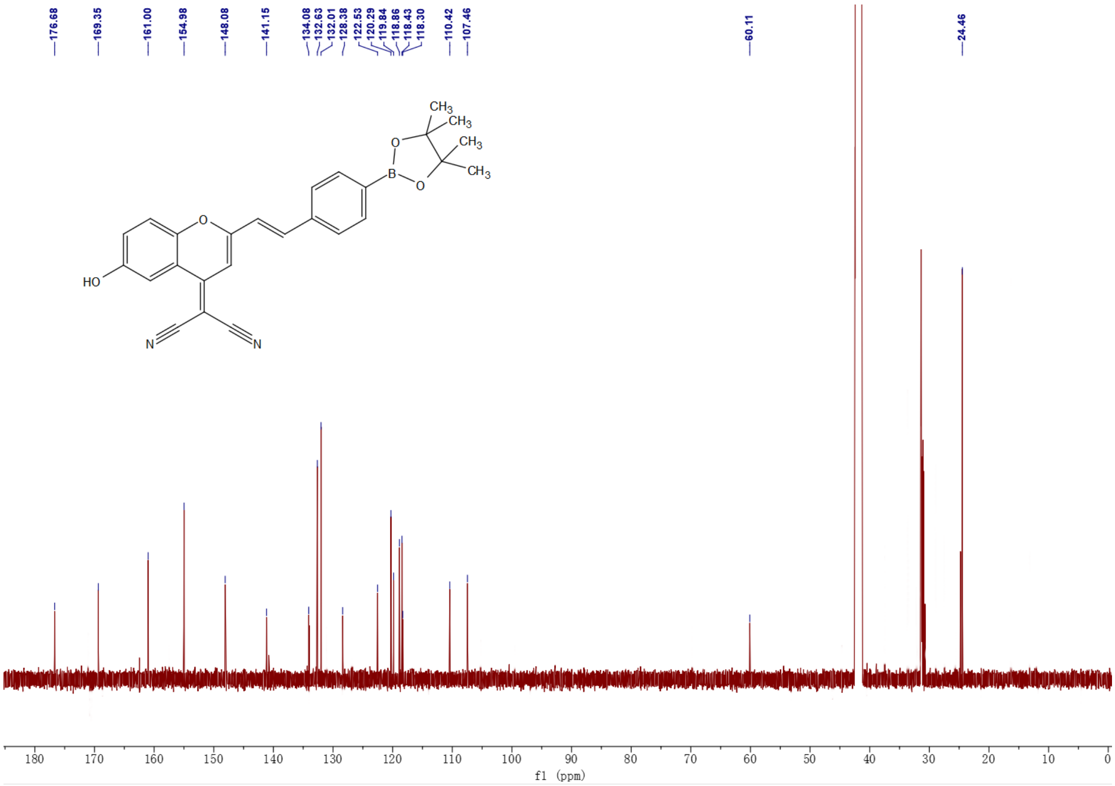

Figure S12. ^{13}C NMR spectrum of **P1** in DMSO-d_6.

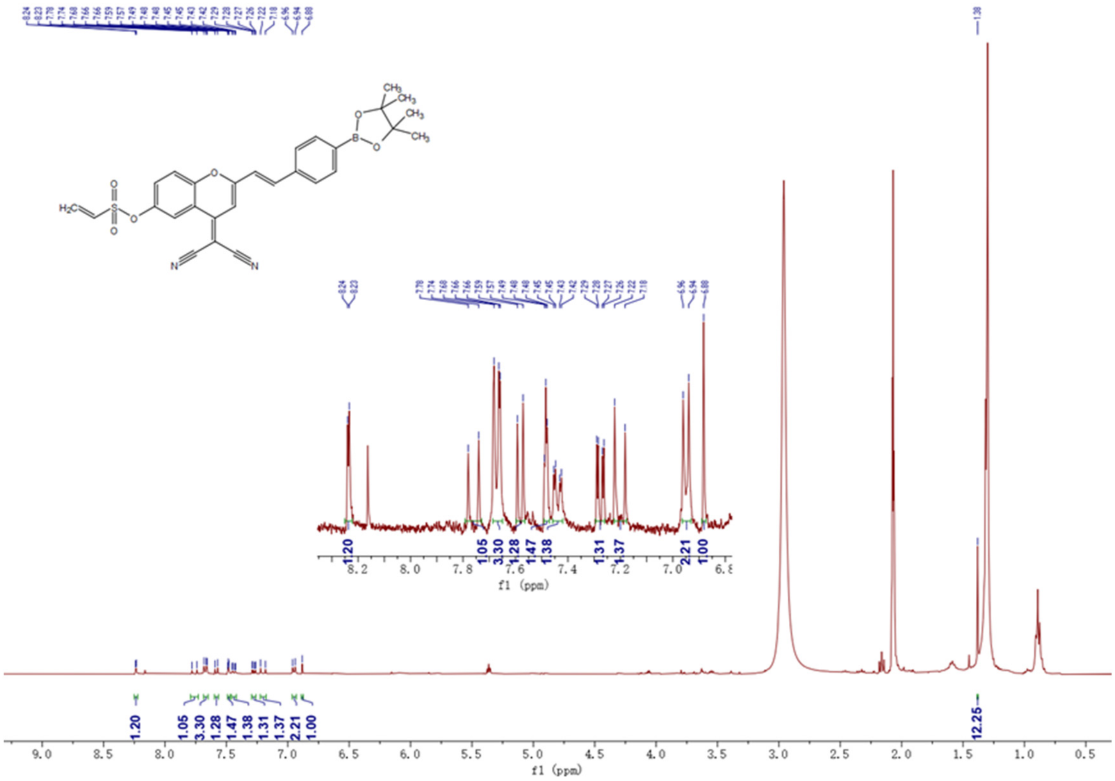

Figure S13. ^{1}H NMR spectrum of **P2** in Acetone-d_6.

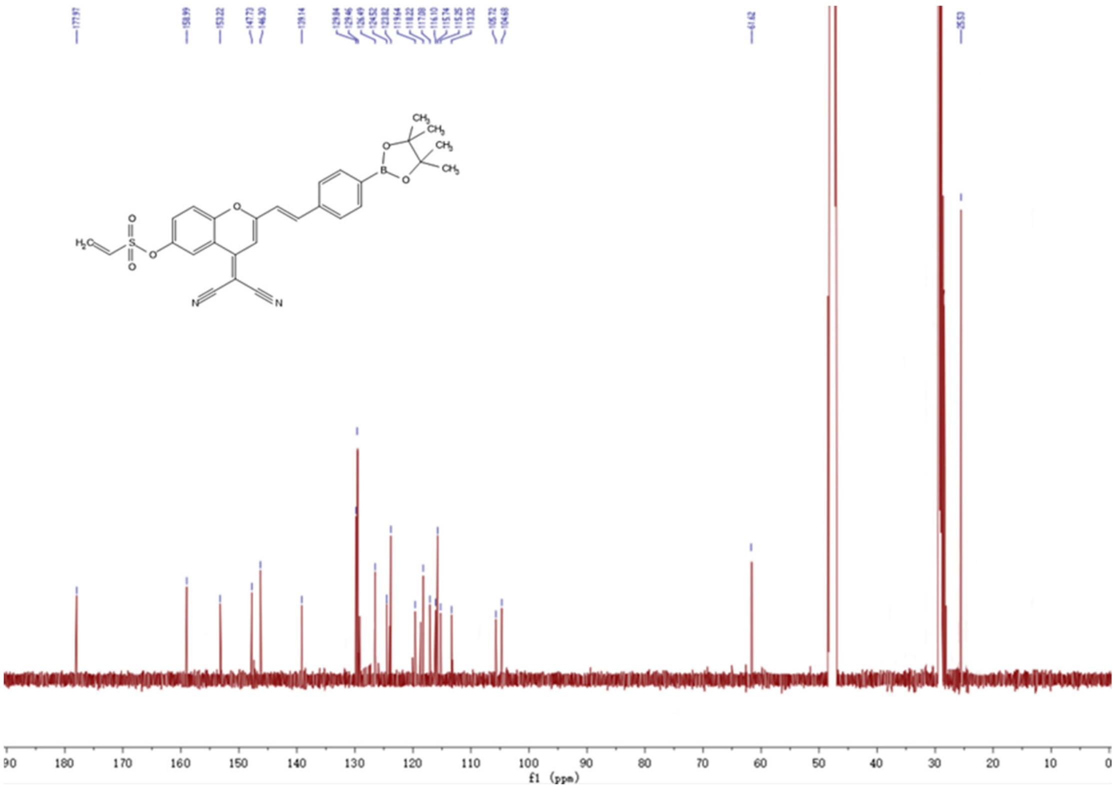

Figure S14. ^{13}C NMR spectrum of **P2** in CD3OD.

9. Assignment of ^{13}C NMR of the probes P1 and P2.

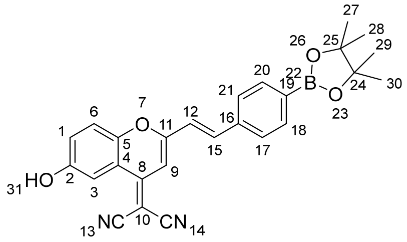

^{13}C NMR (125 MHz, DMSO-d_6) δ 176.68 (s), 169.35 (s), 161.00 (s), 154.98 (s), 148.08 (s), 141.15 (s), 134.08 (s), 132.63 (C-21), 132.63 (C-17), 132.01 (C-20), 132.01 (C-18), 128.38 (s), 122.53 (s), 120.29 (C-14), 120.29 (C-13), 119.84 (s), 118.86 (s), 118.43 (s), 118.30 (s), 110.42 (s), 107.46 (s), 60.11 (s), 24.46 (C-27), 24.46 (C-28), 24.46 (C-29), 24.46 (C-30).

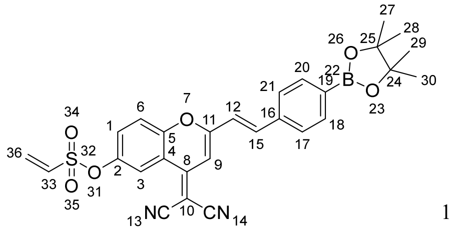

^{13}C NMR (125 MHz, CD_3OD) δ 177.97 (s), 158.99 (s), 153.22 (s), 147.73 (s), 146.30 (s), 139.14 (s), 129.84 (C-21), 129.84 (C-17), 129.46 (C-20), 129.46 (C-18), 126.49 (s), 124.52 (s), 123.82 (s), 119.64 (s), 118.22 (s), 117.08 (s), 116.10 (s), 115.74 (C-14), 115.74 (C-13), 115.25 (s), 113.32 (s), 105.72 (s), 104.68 (s), 61.62 (s), 25.53 (C-27), 25.53 (C-28), 25.53 (C-29), 25.53 (C-30).
